# HSP70 Acetylation Prevents Combined mTORC1/2 Inhibitor and Curcumin Treatment-Induced Apoptosis

**DOI:** 10.3390/molecules23112755

**Published:** 2018-10-24

**Authors:** Seung Un Seo, Kyoung-jin Min, Seon Min Woo, Ji Hae Seo, Taeg Kyu Kwon

**Affiliations:** 1 Department of Immunology, School of Medicine, Keimyung University, 2800 Dalgubeoldaero, Dalseo-Gu, Daegu 704-701, Korea; ssu3885@gmail.com (S.U.S.); kyoungjin.min@gmail.com (K.-j.M.); woosm724@gmail.com (S.M.W.); 2 Department of Biochemistry, School of Medicine, Keimyung University, 2800 Dalgubeoldaero, Dalseo-Gu, Daegu 704-701, Korea; seojihae7@gmail.com

**Keywords:** mTORC1/2, curcumin, PP242, ER stress, HSP70, acetylation

## Abstract

We previously reported that PP242 (dual inhibitor of mTORC1/2) plus curcumin induced apoptotic cell death through lysosomal membrane permeabilization (LMP)-mediated autophagy. However, the relationship between ER stress and apoptotic cell death by combined PP242 and curcumin treatment remains unknown. In the present study, we found that combined PP242 and curcumin treatment induced cytosolic Ca^2+^ release and ER stress. Interestingly, pretreatment with the chemical chaperones (TUDCA and 4-PBA) and knockdown of CHOP and ATF4 by siRNA did not abolish combined treatment-induced apoptosis in renal carcinoma cells. These results suggest that combined treatment with mTORC1/2 inhibitor and curcumin induces ER stress which is not essential for apoptotic cell death. Furthermore, overexpression of HSP70 significantly inhibited PP242 plus curcumin-induced LMP and apoptosis, but the protective effect was abolished by K77R mutation of acetylation site of HSP70. Taken together, our results reveal that regulation of HSP70 through K77 acetylation plays role in combined PP242 and curcumin treatment-induced apoptosis.

## 1. Introduction

mTOR play a critical role in cancer cell growth and is a promising target for cancer therapy. mTOR exists two complexes known as mTOR complex 1 (mTORC1) and mTOR complex 2 (mTORC2) [1]. The efficacy of the mTORC1 inhibitors for cancer therapy is limited, because they suppress the mTORC1-dependent negative feedback loop [1]. Thus, inhibitors for targeting both mTORC1/2 would seem to be necessary to cancer therapy. Although mTORC1/2 inhibitors induce apoptosis in some of cancer cells [2,3], transient inhibition of mTORC or activation of survival signaling prevents anti-cancer effects of mTORC1/2 inhibitor [4,5]. PP242 is developed as an ATP-competitive selective dual inhibitor of mTORC1/2 [6]. In previous study, PP242 alone did not alter cell viability in renal carcinoma Caki cells [7]. However, combined treatment with PP242 and curcumin, which is a dietary polyphenol [8], induces apoptosis through lysosomal membrane permeabilization (LMP)-mediated autophagy. Combined treatment increases cytosolic Ca^2+^ levels, which is a critical signaling molecule for induction of LMP [7]. 

The physiological function of endoplasmic reticulum (ER) and the ER protein-folding environment are impaired, results in induction of ER stress. To relieve ER stress, cells turn on the unfolded protein response (UPR). UPR is mediated by inhibition of protein translation, degradation of misfolding proteins, and induction of molecular chaperones. However, if ER stress is prolonged to the extent that UPR is unable to recover, UPR is turned into apoptotic pathway [9]. Disturbance of Ca^2+^ levels plays a critical for induction of ER stress, resulted in cell death [10]. The heat shock protein (HSP) 70 chaperone plays an important role in protein homeostasis against ER stress condition and protects ER stress-induced apoptotic cell death [11]. In addition to inhibition of ER stress, HSP70 can directly bind to pro-apoptotic protein Bax and Apaf-1, and then interfere with mitochondrial localization [12,13], and HSP70 in lysosome enhances stabilization of lysosome function and allows for autophagy [14,15].

In the current study, we investigated the effect of combined PP242 and curcumin treatment on ER stress induction and the function role of HSP70 acetylation in combined treatment-induced apoptotic cell death in human renal carcinoma cells.

## 2. Results

### 2.1. Combined mTORC1/2 Inhibitor and Curcumin Treatment Induces Endoplasmic Reticulum (ER) Stress

**Figure 1:**
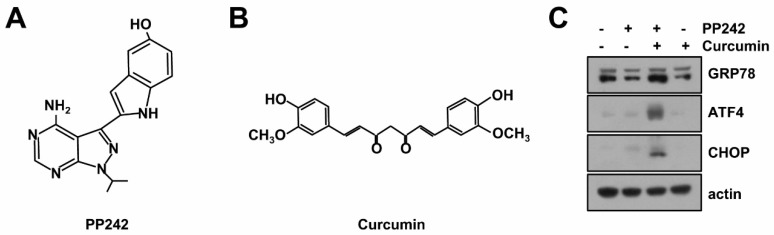
Effects of combined PP242 and curcumin treatment on expression of ER stress marker proteins. Structures of PP242 (**A**) and curcumin (**B**). (**C**) Human renal carcinoma Caki cells were treated with 0.5 µM PP242 in the presence or absence of 20 µM curcumin for 9 h. The protein levels of GRP78, ATF4, CHOP and actin were determined by western blotting. The level of actin was used as a loading control.

We previously reported that PP242 [16], an inhibitor of mTORC1 and mTORC2, and curcumin [17] induced apoptosis through lysosomal membrane permeabilization (LMP)-mediated lysophagy [7] (Figure 1A,B). When combined treatment induced LMP, Ca^2+^ was released from ER and calcium chelators (BAPTA-AM and EGTA-AM) blocked LMP and apoptosis [7]. However, combined treatment-induced ER stress remains unknown. Therefore, we elucidated the effect of combined treatment on induction of ER stress. Combined PP242 and curcumin treatment increased expression of ER stress marker proteins (GRP78, ATF4, and CHOP). However, single treatment of PP242 and curcumin did not induce up-regulation of ER stress marker proteins (Figure 1C). 

### 2.2. Combined Treatment Increases Cytosolic Ca^2+^ Levels

**Figure 2:**
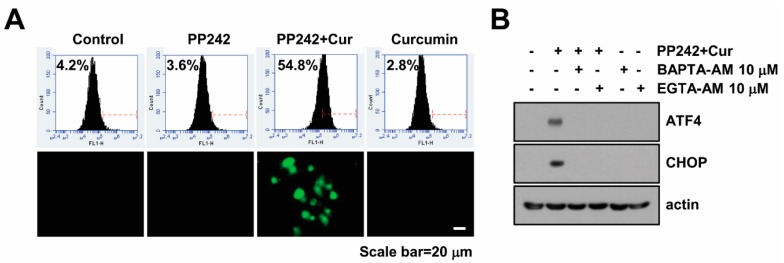
Combined PP242 and curcumin treatment induces intracellular Ca^2+^ release in Caki cells. (**A**) Caki cell were treated with 0.5 µM PP242 in the presence or absence of 20 µM curcumin for 6 h. Cells were loaded with Flou-4/AM fluorescent dye, and flow cytometry (up panel) or fluorescence microscope (low panel) was used to detect calcium levels. (**B**) Caki cells were pre-treated with 10 µM BAPTA-AM and 10 µM EGTA-AM for 30 min and were then treated with PP242 plus curcumin for 9 h. Western blotting was used to detect protein levels of ATF4, CHOP and actin.

Since increase of intracellular Ca^2+^ levels induces ER stress [18], we investigated whether Ca^2+^ levels is correlated with induction of ER stress in PP242 plus curcumin-treated cells. As shown in Figure 2A, PP242 alone and curcumin alone did not increase Fluo-4/AM fluorescence intensities, but combined PP242 and curcumin treatment significantly increased intracellular Ca^2+^ levels within 6 h. In addition, combined treatment increase Fluo-4/AM staining intensities using fluorescence microscopy (Figure 2A). As shown in Figure 1C, PP242 alone and curcumin alone did not induce ER stress, and these results are matched in Ca^2+^ levels (Figure 2A). To further identify the effect of the increased intracellular Ca^2+^ levels on ER stress induction, the cells were incubated with calcium chelators (EGTA-AM and BAPTA-AM). Both chelators inhibited up-regulation of ATF4 and CHOP protein expression in PP242 plus curcumin-treated Caki cells (Figure 2B). These results suggested that combined PP242 and curcumin treatment induces ER stress through the increase of intracellular Ca^2+^ levels. 

### 2.3. Combined PP242 and Curcumin-Induced ER Stress Does Not Affect Cell Death

**Figure 3:**
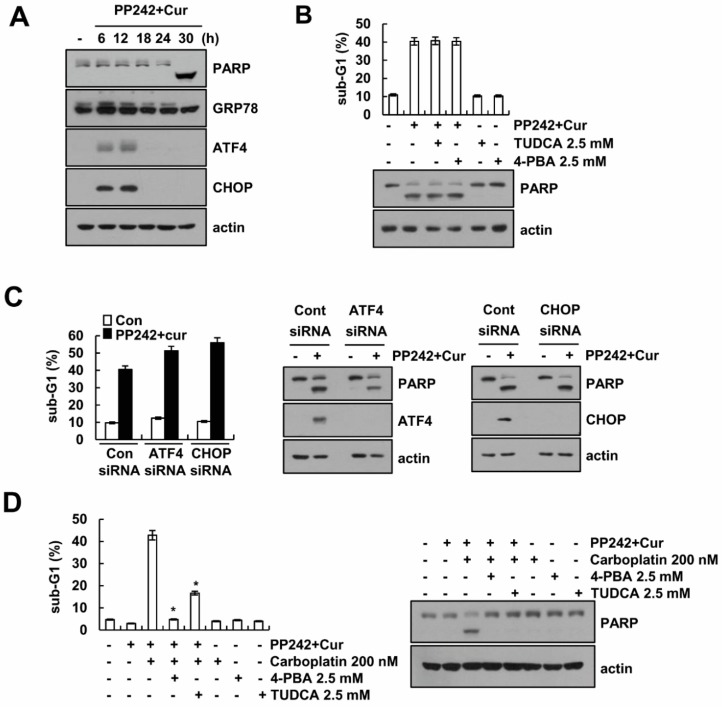
Induction of ER stress is not associated with PP242 plus curcumin-induced apoptosis. (**A**) Caki cells were treated with 0.5 M PP242 plus 20 M curcumin for the indicated time points. (**B**) Caki cells were pre-treated with 2.5 mM TUDCA and 2.5 mM 4-PBA for 30 min and then added with 0.5 µM PP242 plus 20 µM curcumin for 30 h. (**C**) Caki cells were transiently transfected with siRNA against control, ATF4 and CHOP. After 24 h, cells were treated with 0.5 µM PP242 plus 20 µM curcumin for 30 h. (**D**) Caki cells were pre-treated with 2.5 mM TUDCA and 2.5 mM 4-PBA for 30 min and then treated with 0.5 µM PP242 plus 20 µM curcumin in the presence or absence of carboplatin 200 nM for 24 h. Flow cytometry was used to detect the sub-G1 fraction, and western blotting was used to detect the protein levels. The values in (**B**–**D**) represent the mean ± SD of three independent samples. * *p* < 0.01 compared to PP242 plus curcumin in the presence of carboplatin.

Induction of ER stress induces apoptotic cell death in multiple cancer cells. Combined PP242 and curcumin treatment induced poly (ADP-ribose) polymerase (PARP) cleavage at 30 h (Figure 3A). ER stress marker proteins were transiently expressed at 6–12 h, and then declined at 18–30 h (Figure 3A). We next examined whether ER stress effect on combined PP242 and curcumin treatment-induced apoptosis. Chemical chaperones (Tauroursodeoxycholic acid (TUDCA) and 4-Phenylbutyric acid (PBA)) inhibits ER stress [19,20], we expected the chemical chaperons could block apoptosis by PP242 plus curcumin. However, chemical chaperons did not inhibit combined PP242 and curcumin treatment-induced apoptosis (Figure 3B). In addition, siRNA-mediated ATF4 or CHOP knockdown did not inhibit PP242 plus curcumin-induced apoptosis and PARP cleavage (Figure 3C). Therefore, transient induction of ER stress by combined treatment might act as trigger to induce sensitivity against anti-cancer drugs. We examined whether combined treatment enhances carboplatin-induced cell death. As shown in Figure 3D, combined treatment alone and carboplatin alone did not increase apoptosis at 24 h, but combined treatment plus carboplatin significantly induced apoptosis and PARP cleavage. Furthermore, combined treatment plus carboplatin induced apoptosis was markedly inhibited by chemical chaperones (Figure 3D). Our results indicate that PP242 plus curcumin-mediated ER stress does not induce apoptotic cell death, but combined treatment acts as sensitizer against anti-cancer drugs. 

### 2.4. HSP70 Acetylation Inhibits PP242 Plus Curcumin-Induced Apoptosis

**Figure 4:**
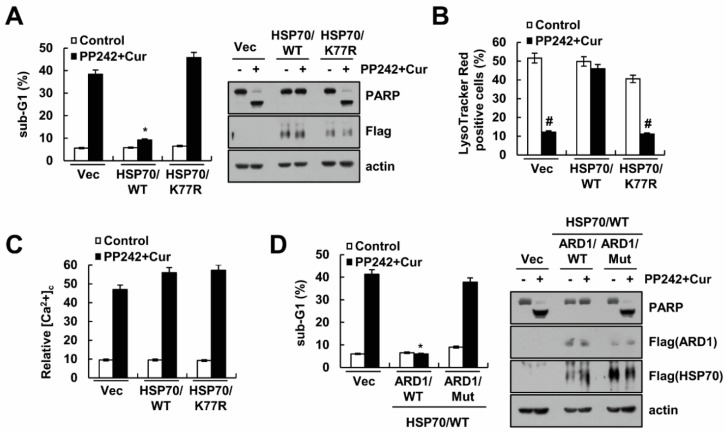
HSP70 acetylation inhibits PP242 plus curcumin-induced apoptosis. (**A**–**C**) Caki cells were transiently transfected with vector, Flag-HSP70 (WT), and mutant Flag-HSP70 (K77R) expression plasmid. After 24 h, cells were treated with 0.5 µM PP242 plus 20 µM curcumin for 30 h (**A**) and 6 h (**B**,**C**). Transfected cells were loaded with LysoTracker Red fluorescent dye (**B**) or Flou-4/AM fluorescent dye (**C**), and fluorescence intensities were detected by flow cytometry. (**D**) Flag-ARD1 WT and dominant negative mutant (Mut) forms were co-expressed with Flag-HSP70 (WT) in Caki cells. After 24 h, cells were treated with 0.5 µM PP242 plus 20 µM curcumin for 30 h. Flow cytometry was used to detect the sub-G1 fraction, and western blotting was used to detect the protein levels of PARP, Flag and actin. The values in (**A**–**D**) represent the mean ± SD of three independent samples. * *p* < 0.01 compared to PP242 plus curcumin-treated Vec. ^#^ *p* < 0.01 compared to the control.

We next investigated whether acetylation of HSP70 play roles in PP242 plus curcumin-induced apoptosis. Acetyltransferase arrest defective (ARD) 1-mediated HSP70 acetylation at K77 modulates stress-induced protein refolding and degradation [21]. Ectopic expression of HSP70 markedly inhibited combined PP242 and curcumin treatment-induced apoptosis, PARP cleavage, and LMP (Figure 4A,B). However, K77R mutant of HSP70 did not inhibit combined treatment-induced apoptosis and LMP (Figure 4A,B). Interestingly, HSP70 wild-type (WT) and K77R mutant did not effect on combined treatment-induced Ca^2+^ release (Figure 4C). To further confirm the relevance of ARD1 in the functional role of HSP70 acetylation, ARD1 WT and a dominant-negative (DN) mutant were co-transfected with HSP70 constructs in Caki cells, and apoptosis and PARP cleavage were assessed after combined PP242 and curcumin treatment. DN mutant ARD1 abolished the protective effect of HSP70 WT (Figure 4D), suggesting that ARD1-mediated HSP70 acetylation contributes to the attenuation of apoptotic cell death in combined PP242 and curcumin treated cells. 

## 3. Discussion

In the present study, we demonstrated that combined PP242 and curcumin treatment induced cytosolic Ca^2+^ release from ER, resulted in induction of ER stress. Induction of ER stress and upregulation of CHOP and ATF4 expression by ER stress were not associated with combined treatment-induced apoptosis in renal carcinoma cells. Interestingly, acetylation of HSP70 prevented combined PP242 and curcumin treatment-induced apoptosis. 

Recently, novel inhibitors of mTORC1/TORC2 are in clinical development with the aim of complete blockade of mTOR complexes and avoidance of the compensatory activation of Akt [22]. We reported that curcumin enhances PP242-induced apoptosis through Bax activation and down-regulation of Bcl-2 and Mcl-1 protein expression [7]. Furthermore, PP242 plus curcumin induces autophagy-mediated apoptosis by downregulation of Rictor and Akt in renal carcinoma cells [7]. However, combined PP242 and curcumin treatment-induced ER stress remains unclear. Our data indicated that PP242 plus curcumin induced ER stress response, but it did not induce apoptotic cell death. As shown in Figure 3A, combined treatment transiently induced up-regulation of ER stress marker proteins, but cleavage of PARP and apoptosis were detected at 30 h. In addition, CHOP siRNA and chemical chaperones did not abolish combined PP242 and curcumin treatment-induced apoptosis (Figure 3B,C). Cancer cells can adapt mild ER stress, but prolong and severe ER stress induces various types of cell death [23]. Transient induction of ER stress by combined treatment might act as trigger to induce sensitivity against anti-cancer drugs (Figure 3D). Therefore, the unfolded protein response is a helpful target for anticancer therapeutics. 

Recently, curcumin is classified as a PAINS (pan-assay interference compounds) and an IMP (invalid metabolic panaceas), and Nelson et al. suggests potential guidance about curcumin research, which could reduce false activity of curcumin [24]. In our study, we used curcumin alone or combined treatment with PP242 and curcumin as same concentrations, and curcumin alone has no effect on any experiments. Therefore, the effect of curcumin was not induced by interference, but we could not rule out the all potential problems of curcumin as a PAINS and IMP. 

The molecular chaperon HSP70 is highly expressed in most tumor cells. We also previously reported that ectopic expression of HSP70 inhibited cathepsin S inhibition- and PP242 plus curcumin-induced LMP and apoptotic cell death [7,25]. HSP70 is known as a chaperone, as well as an endogenous inhibitor of LMP [14]. Functional role of protein refolding and degradation by HSP70 is regulated by cooperation with co-chaperones such as Hop and CHIP [26,27]. In addition, activity of HSP70 is also modulated by protein modification such as acetylation and phosphorylation [21,28]. Recently, Park et al. reported that HSP70 acetylation prevented caspase-dependent/independent apoptosis and autophagic cell death [29]. ARD1-mediated HSP70 acetylation at K77 residue balances stress-induced protein refolding and degradation [21]. In our study, ectopic expression of HSP70 did not inhibit Ca^2+^ release, whereas induction of LMP is prevented in PP242 plus curcumin-treated cells (Figure 4B,C). Furthermore, ectopic expression of K77R mutant form of HSP70 did not inhibit combined PP242 and curcumin treatment-induced apoptosis and PARP cleavage, as well as induction of LMP (Figure 4A). Therefore, HSP70 blocked PP242 plus curcumin-induced apoptosis through inhibition of LMP. Interestingly, co-transfection of the DN mutant ARD1 prevented HSP70 acetylation-induced inhibition of apoptosis and PARP cleavage (Figure 4D). These data suggest the possibility that acetylation of HSP70 (K77) by ARD1 might be involved in inhibition of LMP. Another possible mechanism of HSP70-mediated cell death inhibition is change of binding proteins. The K77 site is located at nucleotide binding domain (NBD) of HSP70 [21]. Acetylation site K77 in NBD may modulate the HSP70 conformational change which is a critical role in its protein domain interaction with counter proteins [21]. Therefore, there is a possibility that combined PP242 and curcumin-induced acetylation of HSP70 switch to interaction with co-chaperones in apoptotic condition. We need further experiments to identify the mechanism of acetylation of HSP70.

Collectively, these results reveal that combined PP242 and curcumin treatment induces ER stress which is not involved in apoptotic cell death. The acetylation of HSP70 plays a critical role in combined PP242 and curcumin-induced apoptosis.

## 4. Material and Methods

### 4.1. Cell Culture and Materials

American Type Culture Collection supplied human renal carcinoma Caki cells (Manassas, VA, USA), which grown in DMEM supplemented with 10% FBS and 100 µg/mL gentamycin. Caki cells were tested for mycoplasma contamination. Cells were authenticated by standard morphologic examination using microscopy (Carl Zeiss, Jena, Germany). PP242 and curcumin were purchased from Selleckchem (Huston, TX, USA) and Biomol (Plymouth Meeting, PA, USA), respectively. Calbiochem supplied EGTA-AM (San Diego, CA, USA). Tocris supplied carboplatin (Bristol, UK). Santa Cruz Biotechnology supplied anti-ATF4 antibody, TUDCA and siRNA (CHOP and ATF4), and Cell Signaling Technology supplied anti-PARP and anti-CHOP antibodies (Beverly, MA, USA). Enzo life science supplied anti-GRP78 antibody (Farmington, NY, USA). Bioneer supplied the green fluorescent protein (GFP; control) siRNA (Daejeon, Korea). Sigma Chemical Co. supplied other reagents and anti-actin and anti-Flag antibodies used in our study (St. Louis, MO, USA).

### 4.2. Western Blot Analysis and Flow Cytometry Analysis

Whole cell lysates were collected as described previously using modified RIPA [30,31,32]. We performed the western blotting and flow cytometry analysis as described in our study [33].

### 4.3. Intracellular Ca^2+^ Detection and Measurement of LMP

To detect Ca^2+^ levels, cells were harvested and resuspended in PBS containing 2 µM of Fluo-4/AM (Molecular Probes, Invitrogen, Carlsbad, CA, USA) for 45 min. After centrifuges, the cells were resuspended in PBS for FACS acquisition (BD Biosciences, San Diego, CA, USA). To monitor of lysosomal destabilization, we used LysoTracker Red. Cells were stained with LysoTracker Red (2.5 µM) and washed twice with PBS, and fluorescence was analyzed by flow cytometer. 

### 4.4. Statistical Analysis

Data in our study were analyzed by a one-way ANOVA and post-hoc comparisons (Student-Newman-Keuls) of the Statistical Package for Social Sciences 22.0 software (SPSS Inc.; Chicago, IL, USA).

## Abbreviations

CHX	Cycloheximide
ROS	Reactive Oxygen Species
ER	Endoplasmic Reticulum
UPR	Unfolded Protein Response
CHOP	CCAAT-Enhancer-Binding Protein Homologous Protein
ATF4	Aactivating Transcription Factor 4
PARP	Poly (ADP-ribose) Polymerase
